# Medaka villin 1-like protein (VILL) is associated with the formation of microvilli induced by decreasing salinities in the absorptive ionocytes

**DOI:** 10.1186/1742-9994-11-2

**Published:** 2014-01-13

**Authors:** Chao-Kai Kang, Tsung-Han Lee

**Affiliations:** 1 Department of Life Sciences, National Chung-Hsing University, Taichung 402, Taiwan; 2 Department of Biological Science and Technology, China Medical University, Taichung 404, Taiwan

**Keywords:** Microvilli, Ionocyte, Villin 1-like protein, Medaka, Gill

## Abstract

**Introduction:**

Villin 1 is an actin-regulatory protein involved in the formation of microvilli of mammalian enterocytes. The microvilli, finger-like protrusions, are more abundant on the apical surfaces of gill ionocytes in various freshwater (FW) teleosts than in seawater (SW) fishes. However, the plasticity in the mechanisms of microvillus formation in the gill ionocytes are poorly understood, and the actin-regulatory proteins involved in the formation of microvilli have not been identified in fishes. The present study used the euryhaline medaka (*Oryzias dancena*) as a model to explore the role of a homolog of villin 1 in the actin-organization of cellular morphologies induced by decreasing salinities.

**Results:**

By ultrastructural observation, there are numerous actin filaments organized on the apical cortex of ion-absorptive ionocytes in the FW-acclimated medaka. From gills of the euryhaline medaka, we have identified the VILL sequence. The phylogenetic tree and functional domains suggest that VILL is the homolog of villin 1 in fishes. Immunofluorescence using a specific antibody revealed that VILL was specifically localized to the apical region of gill ionocytes along with microvilli in the FW medaka, but not in SW fish. The expression levels of *Odvill* mRNA and VILL protein were higher in the gills of the FW individuals than in the SW group and were induced when fish were transferred from SW to FW. A morpholino oligonucleotide for VILL knockdown eliminated the apical protrusions of ionocytes and pavement cells in the trunk epithelia of embryos.

**Conclusions:**

From a novel aspect of cytoskeletal functions, our findings highlighted the important role of VILL protein in the ionoregulation of aquatic vertebrates in response to different osmotic challenges. This study is the first to show that the expression of VILL is associated with the formation of microvilli in the absorptive ionocytes of a euryhaline fish. Loss-of-function experiments showed that the distribution of VILL may represent the molecular link between the cytoskeletal organization and cellular morphology of the absorptive ionocytes during hypoosmotic adaptation in aquatic vertebrates.

## Introduction

Teleostean fishes are aquatic osmoregulators that can maintain plasma homeostasis in environments with many fluctuating components. The gill, which is exposed to the external environment, is one of the major osmoregulatory organs in fishes. In recent years, several review articles have illustrated the regulatory systems of crucial ion transporters in gill ionocytes, also called mitochondrion-rich cells or chloride cells [1-8]. Ionocytes are polar, with apical and basolateral membranes that face the external media and internal plasma, respectively [3]. Gill ionocytes express Na^+^, K^+^-ATPase (NKA) on the basolateral membrane, providing the driving force that enables other ion transporters or channels on the plasma membrane to absorb ions in fresh water (FW) and to secrete ions into seawater (SW) [1-8]. According to ultrastructural observations by scanning electron microscopy (SEM) and transmission electron microscopy (TEM), two typical types of gill ionocytes can be identified by their apical morphologies and the acclimated salinities. In various teleosts, the SW-type cells exhibit deeply invaginated surfaces with smaller orifices, while the FW-type ionocytes generally show large openings and membrane surfaces with finger-like cytoplasmic protrusions, similar to microvilli. The microvilli of gill ionocytes serve to enlarge the apical surface area to enhance ion absorption in the FW teleosts [5,8-13].

The gill ionocytes modulate the expression of ion transporters and remodel their cellular morphologies to regulate ion concentrations between their blood and the environment. The apical morphologies of ionocytes in fish gills transform and change in size or shape for different ionoregulatory functions in euryhaline teleosts when exposed to environments with various salinities or concentrations of ions [5,10,12,13]. This plasticity involves the appearance and disappearance of microvilli upon exposure to decreasing or increasing environmental salinities, respectively [12,13]. In the epithelial cells of mammals, microvilli are composed of actin filaments that originate from the terminal web of the “cell cortex” (i.e., the cytoplasmic area close to the apical region) [14,15]. The reorganization of actin filaments is associated with the plasma membrane dynamics, which remodel the cell morphology to perform specific functions of epithelial cells [14-16]. According to previous TEM observations, more cytoskeletal filaments were found in the cell cortex rather than the cytoplasm of gill ionocytes in fishes [10,17-19]. On the other hand, Wong et al. [20] reported that the ionocytes express actin protein in the Japanese eel. These findings imply that the actin cytoskeleton is involved in the formation of microvilli in the apical membrane of gill ionocytes. To date, however, there is no known actin-regulatory molecule that governs the remodeling of the apical microvilli in fish ionocytes in response to changes in environmental salinities.

Villin 1, an actin-regulatory protein, was first isolated from chicken intestinal brush border [21]. Based on the structural and functional domains of villin 1, its gelsolin and headpiece domains exhibit actin-capping, -severing, -nucleating, and -bundling for the reorganization of actin filaments [22-24]. The villin 1 of mammals can polymerize G-actin and depolymerize F-actin to influence cell morphology, migration, invasion, and survival [24]. Human villin 1 is expressed prominently in renal, gastrointestinal, and urogenital epithelial cells, where it is localized to the microvillar core and the terminal web of apical structures [24-26]. Villin 1 plays roles in the initiation, organization, and formation of microvilli, which absorb and reabsorb nutrients in enterocytes and proximal tubule cells [27]. Different levels of villin 1 expression influence the microvillus structures of two cell lines [28,29]. Thus, villin 1 is an important factor in the cell morphologies of mammals. In the fish cells, however, no homolog of villin 1 has been identified.

The brackish medaka (*Oryzias dancena*) is a small euryhaline fish with good salinity tolerance [30,31]. Our previous studies have demonstrated the mechanisms of ionoregulation in gill ionocytes of this medaka [13,32-35]. The present study verified the microvillus ultrastructures on the apical surfaces of ionocytes in gills of the FW-acclimated medaka by SEM and TEM. When searching for a homolog of villin 1 that is associated with the organization of cytoskeletal filaments in FW-type ionocytes, we identified the gene sequence of villin 1-like protein (VILL) from the euryhaline medaka, detected the gene and protein expression of VILL in gill ionocytes with different microvillus ultrastructures, and demonstrated the function of VILL in determining the apical morphologies of embryonic ionocytes. These findings suggest an important role for VILL in a novel aspect of cytoskeletal function in the ionocytes of aquatic vertebrates in response to different osmotic challenges.

## Results

### Finger-like protrusions of ionocytes in the FW medaka

**Figure 1:**
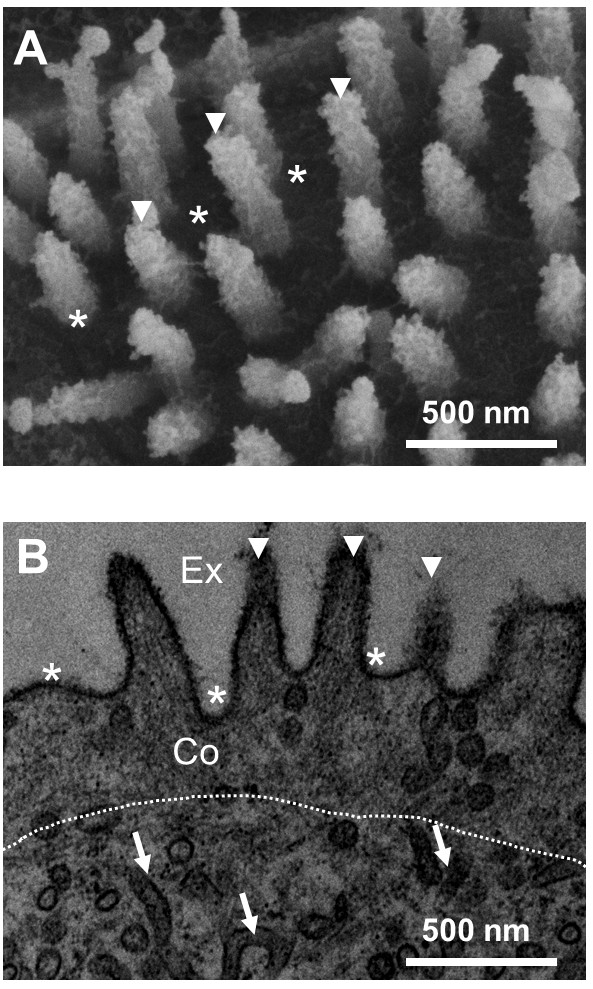
**The ultrastructures of microvilli were observed (50,000×) in the apical regions of FW-type ionocytes under scanning electronic microscope (SEM; A) and under transmission electronic microscope (TEM; B).** The cell cortex (Co) with numerous cytoskeletal filaments was identified by its different electron density (white dotted line) in the cell cortex. The microvilli (arrowheads) containing cytoskeletal filaments extended from the cell membrane (asterisks). The arrows indicated the tubular system in the cytoplasm of the ionocyte. Ex, external environment.

In the FW medaka, SEM and TEM observations showed numerous finger-like protrusions extending from the cell membrane in the apical opening of the ionocytes (Figure 1). The diameters and lengths of the protrusions were approximately 100 and 500 nm, respectively. Close to the apical membrane, there was a region rich in cytoskeletal filaments with high electron density. No membrane-formed tubule (i.e., the tubular system) was found in this region like the other part of the cytoplasm. The finger-like protrusions exhibited longitudinally arranged filaments (Figure 1B). The microvilli on apical surfaces were mainly found in ionocytes of FW-acclimated medaka but not in the hole-type ionocytes of SW-acclimated individuals (Additional file 1: Figure S1). However, 50%-SW-acclimated medaka exhibited some hole-type ionocytes with microvilli but other hole-type ionocytes without microvilli (Additional file 1: Figure S2).

### Cloning and characterization of villin-like (VILL) protein from the fish gill

**Figure 2:**
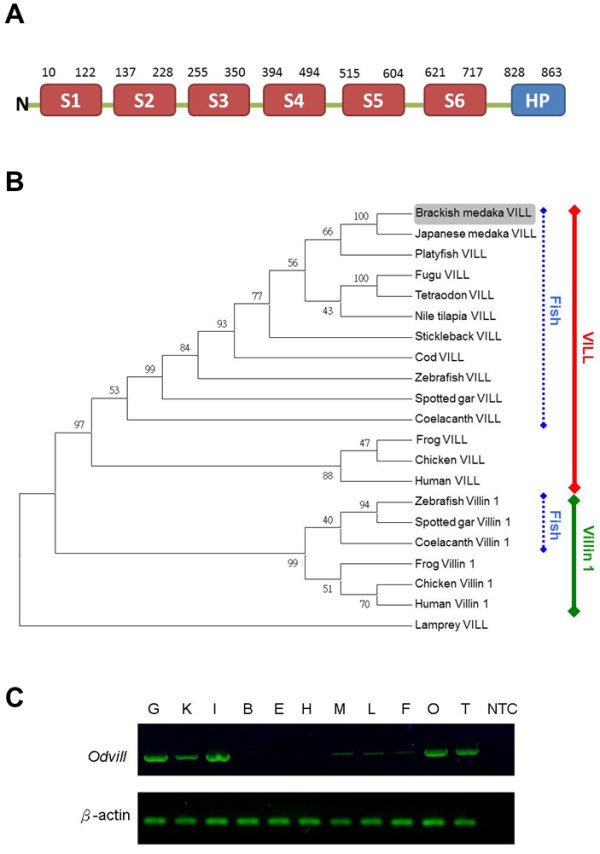
**The sequence of VILL was identified in the brackish medaka. (A)** Schematic representation of the domains of the villin 1-like (VILL) protein sequence deduced from *Odvill*. Six putative gelsolin segments (S1-S6) were indicated (red box). The villin headpiece domain (HP) was represented by a blue box. **(B)** Phylogenic analysis of full-length amino acid sequences of villin and VILL from different species from the Ensembl Genome Browser, shown as a phylogenetic tree created by the maximum parsimony method with 1000 bootstrap replicates. Brackish medaka VILL protein was shaded. **(C)** Tissue distribution of *Odvill* detected by RT-PCR in the brackish medaka. The β-actin was used as an internal control. B, brain; E, eye; F, fin; G, gill; H, heart; I, intestine; K, kidney; L, liver; M, muscle; O, ovary; T, testis; NTC, no-template control.

A 3114 bp full-length *Odvill* cDNA (KF011977) encoding an 864 amino acid protein was isolated from gills of the brackish medaka (Additional file 1: Figure S3). The cDNA contained 118 bp of 5′ UTR and 404 bp of 3′ UTR. There were six gelsolin domains and a headpiece domain in the deduced VILL protein of the brackish medaka (Figure 2A). The present study surveyed databases of the 11 fish species from the ensemble genome browser (Table 1). According to the phylogenetic tree (Figure 2B), the homologous proteins of the vertebrates could be divided into two subclasses: villin 1 and VILL. The gene of *vill* was found in all examined vertebrates. However, the *villin 1* was found in three fishes (among the 11 surveyed species) and the frog, chicken, and human. In these vertebrates, the specific sequences of villin 1 were identified, and their differences with VILL were illustrated in the Additional file 1: Figure S3. The derived amino acid sequence of VILL from the brackish medaka showed 89% identity to that of the Japanese medaka and about 63-73% identity to those of the other fishes, 47-58% identity to the VILL clade of other higher vertebrates, and 46-55% identity to members of the villin 1 clade. RT-PCR analysis of 11 organs revealed that *Odvill* (735 bp) were expressed mainly in the gill, kidney, intestine, ovary, and testis (Figure 2C). In addition, lower levels of *Odvill* were found in the muscle, liver, and fin.

**Table 1:** The gene and protein information of VILL and villin 1 in 11 fishes from the Ensembl Genome Browser

**Species**	**Gene name**	**Gene number**	**Transcript number**	**bp**	**Protien number**	**a. a**	**Genome location**
1. Japanese medaka	VILL	ENSORL00000009593	ENSORL00000012032	2589	ENSORL00000012031	862	Chromosome 20: 16,306,576-16,314,374 forward strand
2. Tetraodon	VILL	ENSTNIG00000012740	ENSTNIT00000015922	2511	ENSTNIP15715	836	Chromosome 6: 4,018,315-4,024,446 forward strand
3. Fugu	VILL	ENSTRUG00000017516	ENSTRUT00000045055	2607	ENSTRUP00000044903	868	scaffold_326: 94,677-100,136 reverse strand
4. Stickleback	VILL	ENSGACG00000003464	ENSGACT00000004567	2966	ENSGACP00000004553	826	group XXI: 8,029,734-8,038,141 forward strand
5. Cod	VILL	ENSGMOG00000010755	ENSGMOT00000011828	2409	ENSGMOP00000011514	803	GeneScaffold_827: 84,123-98,980 forward strand
6. Nile tilapia	VILL	ENSONIG00000005814	ENSONIT00000007327	6699	ENSONIP00000007322	908	Scaffold GL831138.1: 315,520-331 forward strand
7. Platyfish	VILL	ENSXMAG00000009353	ENSXMAT00000009424	7424	ENSXMAP00000009410	845	Scaffold JH556681.1: 954,240-974,404 reverse strand
8. Zebrafish	Villin 1	ENSDARG00000040466	ENSDART00000059228	3265	ENSDARP00000059227	834	Chromosome 9: 45,978,151-46,015,871 reverse strand
	VILL	ENSDARG00000001909	ENSDART00000048940	2809	ENSDARP00000048939	850	Chromosome 24: 21,027,155-21,049,407 reverse strand
9. Spotted Gar	Villin 1	ENSDAR00000059227_1	ENSDARP00000059227_1	2385	ENSDARP00000059227_1	795	Chromosome LG12: 19,340,570-19,351,792 reverse strand
	VILL	ENSDARP00000048939_1	ENSDARP00000048939_1	2316	ENSDARP00000048939_1	772	Chromosome LG9: 25,680,983-25,696,249 forward strand
10. Coelacanth	Villin 1	ENSLACG00000001107	ENSLACT0000001245	2499	ENSLACP00000001233	832	Scaffold JH128253.1: 10,112-60,018 reverse strand
	VILL	ENSLACG00000006000	ENSLACT00000006819	2226	ENSLACP00000006764	741	Scaffold JH129281.1: 196,217-235,545 forward strand
11. Lamprey	VILL	ENSPMAG00000000	ENSPMA00000000954	3098	ENSPMAP00000000950	870	Scaffold GL477991: 15,745-43,529 reverse strand

### Apical distribution of VILL protein in FW-type ionocytes

**Figure 3:**
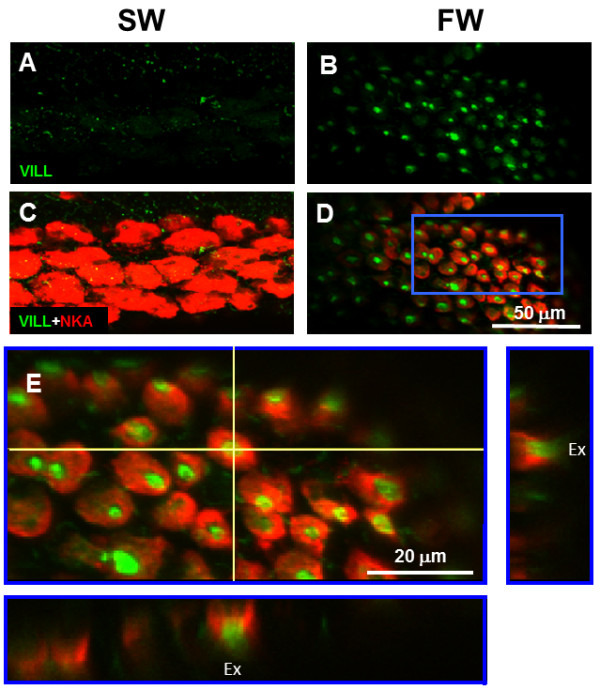
**The VILL located to the apical region of the FW-type ionocytes.** Confocal micrographs of the whole-mount double immunofluorescence staining with anti-VILL (green; **A** and **B**) and anti-NKA antibodies (red) on the afferent sides of gill filaments in the brackish medaka acclimated to FW or SW. The merged images **(C and D)** revealed that the VILL protein was localized to the NKA-immunoreactive (NKA-IR) cells in the FW fish but not in the SW fish. **(E)** The magnified merged 3D image of the FW fish gill revealed VILL signals in the apical region of NKA-IR cells. Ex: external environment.

A polyclonal antibody against the headpiece domain of medaka VILL was produced (Additional file 1: Figure S3). Figure 3 shows the distribution of VILL protein in the afferent epithelia of gill filaments of the FW- and SW-acclimated medaka using the whole-mount double immunofluorescence staining. The merged images (Figure 3C,D) revealed that VILL protein was localized in the NKA immunoreactive (NKA-IR) cells in gills of the FW fish rather than in the SW group. The magnified merged 3D image (Figure 3E) revealed that the VILL signals were localized to the apical region of NKA-IR cells in the gill filaments of FW-acclimated medaka. In addition, double immunofluorescence staining with T4 antibody and VILL antibody showed that T4 signals of the apical Na^+^-K^+^-2Cl^-^ cotransporter were colocalized with VILL signals at the apical region of some ionocytes in the gill filaments of FW-acclimated medaka (Additional file 1: Figure S4).

### Chronic and acute expression of VILL in gills

**Figure 4:**
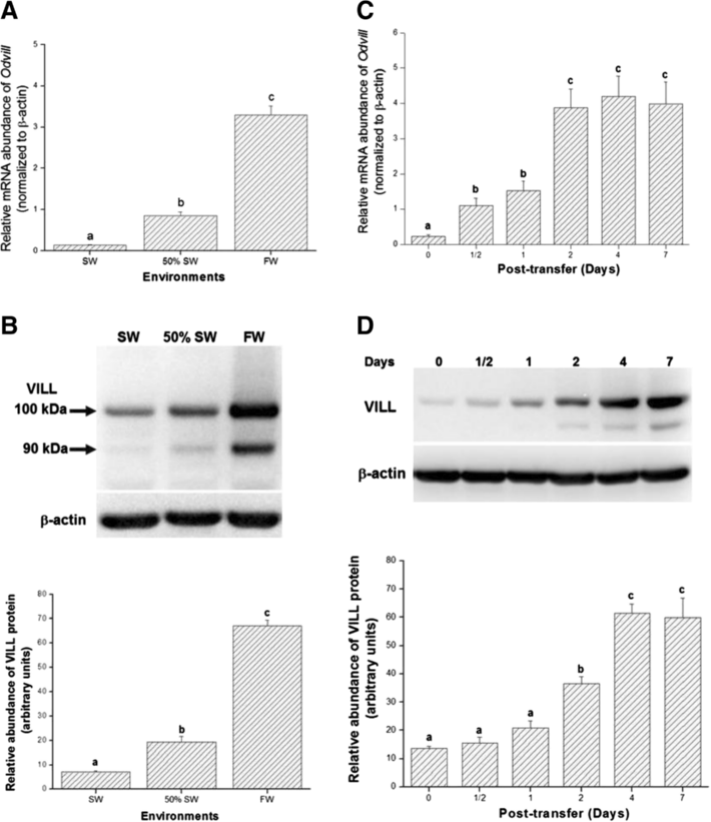
**The expression of gill VILL was induced by the decreasing environmental salinities. (A)** The mRNA levels of *Odvill* in gills of the brackish medaka acclimated to SW, 50% SW, or FW (n=6 for all groups). The mRNA abundance increased with decreasing environmental salinity. **(B)** Representative immunoblot of VILL from gills of the brackish medaka acclimated to FW, 50% SW, or SW, detected with the specific polyclonal antibody. Two immunoreactive bands were found at 100 kDa and 90 kDa. The sum of relative intensities of the two immunoreactive bands of branchial VILL protein was analyzed and compared among the three salinity groups (n=6 for each) to show that the amount of VILL significantly increased with decreasing environmental salinity. **(C)** Dynamic expression of *Odvill* mRNA in gills of the brackish medaka transferred from SW to FW. *Odvill* mRNA were significantly increased to approximately 5-fold 1 day post-transfer and increased to approximately 17-fold 2 days post-transfer compared to baseline (0 day; SW) (n=5 for all groups). **(D)** Representative immunoblot of VILL in gills of the brackish medaka after transfer from SW to FW, as detected by the specific antibody to VILL. Dynamic expression of the VILL protein in gills of the brackish medaka transferred from SW to FW. The abundances of VILL increased gradually in the first 2 days after transfer (3-fold) and increased to 5-fold after 4 days compared to baseline (0 day; SW) (n=5 for all groups). β-actin was used as the loading control. Different letters indicated significant differences (p < 0.05) using Tukey’s multiple comparison test following a one-way ANOVA. The values are means ± S.E.M.

Quantification of branchial *Odvill* mRNA in medaka acclimated to SW, 50% SW, or FW revealed the highest abundance in the FW fish, approximately 6-fold and 24-fold of the expression in 50% SW and SW fish, respectively (Figure 4A). The medaka VILL antibody was used to detect its localization and abundance. Compared to the negative control (immunoblots using the rabbit pre-immune serum; Additional file 1: Figure S5), there were two immunoreactive bands, at 100 kDa and 90 kDa, in the total lysates of gill samples. The major band at 100 kDa was detected in all groups, while the minor band at 90 kDa was found in the FW and 50% SW groups (Figure 4B). Combining the amounts of these two immunoreactive bands, FW fish expressed the highest level of VILL in gills (approximately 3-fold of the level in the 50% SW group and 10-fold of the level in the SW group) (Figure 4B). The amounts of VILL of the 50% SW group were significantly higher (approximately 3-fold) than in the SW fish. Time-course experiments showed increased expression of VILL gene and protein in gills when SW medaka were transferred to FW (Figure 4C,D). The abundance of branchial *Odvill* mRNA increased significantly (approximately 5-fold) at 1/2 day post-transfer. At 2 days after transfer, the *Odvill* mRNA of the FW-exposed fish increased to approximately 17-fold higher than the SW group (Figure 4C). In the immunoblots of the SW-acclimated medaka and the fish transferred to FW for 1/2, 1, 2, 4, or 7 days, gill VILL also displayed a major immunoreactive band at 100 kDa in all groups, while a minor band at 90 kDa was found in the 2-, 4-, and 7-day-transfer groups (Figure 4D). Compared to the SW group, the abundance of VILL in FW-exposed medaka increased significantly (approximately 3-fold) at 2 days post-transfer. The amounts of VILL in the 4- and 7-day-transfer groups were approximately 5-fold higher than in the SW group (Figure 4D).

### VILL-MO inhibited expression of VILL in embryonic ionocytes

**Figure 5:**
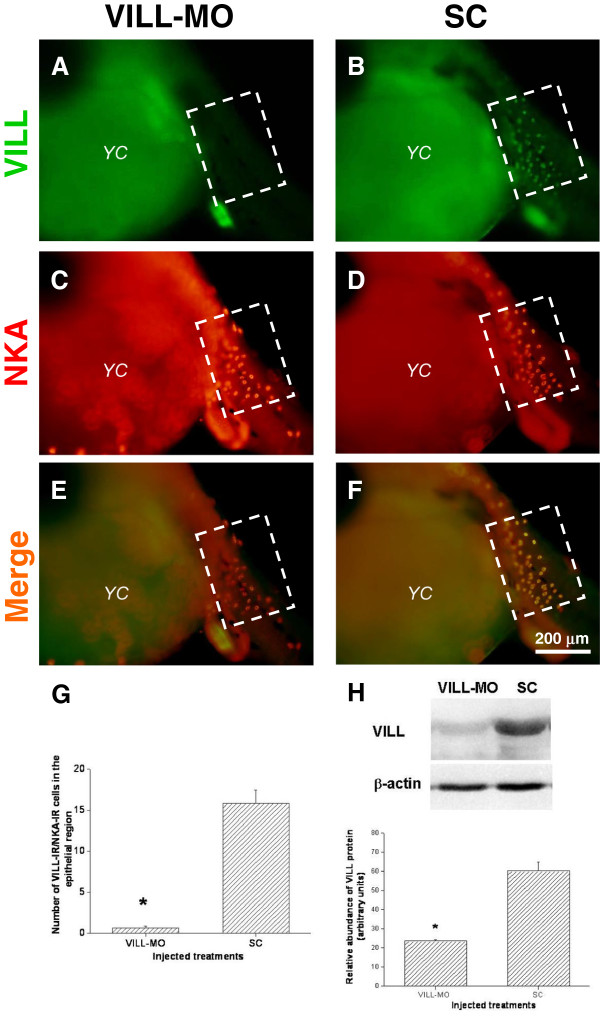
**Knockdown of VILL with a morpholino oligonucleotide (MO) interfered with the protein expression of ionocytes in the trunk epithelia of embryos incubated in FW for 6 dpf.** The embryos injected with VILL-MO **(A, C, and E)** or standard control (SC) oligonucleotide **(B, D, and F)** were whole-mount double-immunofluorescence-stained with anti-VILL (green; **A** and **B**) and anti-NKA (red). The fixed regions for observation were indicated with white box on the lateral side of the posterior trunk. The merge images **(C and D)** revealed that VILL-IR signals were faint in the observed region of VILL-MO embryos compared to the signals of the SC group. The arrows indicated that the VILL-IR signals were found in the guts of both two groups of embryos. YS, yolk sac. **(G)** Average numbers of NKA-IR cells with VILL signals in the fixed region (the white boxes) of the trunk epithelia were quantified and compared between VILL-MO- and SC-injected embryos at 6 dpf (n=12 for all groups). The number of NKA-IR cells with VILL signals of the VILL-MO-injected embryos was significantly lower than in the SC-injected group. **(H)** Representative immunoblot of VILL protein detected with a specific polyclonal antibody in the embryos microinjected with VILL-MO or SC. The molecular mass of the single immunoreactive band was 100 kDa. β-actin was used as the loading control. Relative intensities of immunoreactive bands of VILL in embryos of the VILL-MO and SC groups (n=6 for both groups) were analyzed and compared to show that the amount of VILL in the VILL-MO group was significantly lower than in the SC group. The asterisk indicates significant differences (P <0.05, Student’s t-test). The values are means ± S.E.M.

**Figure 6:**
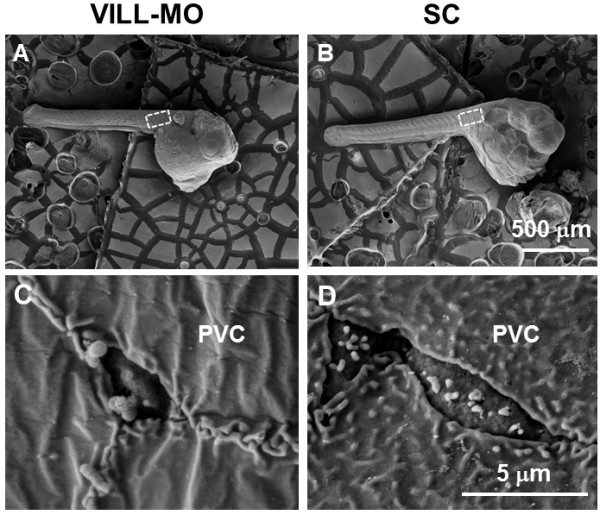
**The morphological effects of VILL gene knockdown with MO were investigated in the trunk epithelia of the FW-incubated embryos at 6 dpf.** Morphologies of the VILL-MO- **(A and C)** and SC-injected **(B and D)** embryos at 6 dpf were compared by SEM. The observed region is indicated by white boxes **(A and B)** in the embryonic trunks. SEM micrographs showed the morphological differences in the apical surfaces of trunk epithelial cells between a VILL-MO-injected embryo **(C)** and a SC embryo **(D)**. The apical surfaces had abnormal morphology, with impaired microvilli, in the trunk ionocytes of the VILL-MO-injected embryo compared to the SC embryo. The apical surfaces of pavement cells (PVC) lacked microridges in the VILL-MO-injected embryo.

The whole-mount double immunofluorescence staining with VILL and NKA antibodies showed the VILL-IR/NKA-IR cells on the posterior trunks of embryo incubated in the FW at 6 dpf (Additional file 1: Figure S6). The magnified merged confocal microscopic 3D images revealed that the VILL signals were localized at both basolateral (compared to NKA) and apical regions of NKA-IR cells. In addition, the circular patterns of VILL signals were also found in the pavement cells adjacent to NKA-IR cells. The medaka embryos were microinjected with VILL-MO to evaluate the morphological changes of trunk ionocytes. Quantification of the number of NKA-IR cells with VILL signals in the fixed region of the posterior trunk (Figure 5A-F) revealed that there was no significant difference in the number of NKA-IR cells between the VILL-MO- and SC-injected groups (VILL-MO vs. SC: 28.3 ± 1.4 vs. 27.1 ± 1.7). Meanwhile, the number of NKA-IR cells with VILL signals in the VILL-MO-injected group was significantly lower (approximately 24-fold) than in the SC-injected group (Figure 5G). In addition, strong VILL-IR signals were found in the guts of VILL-MO and SC-injected embryos (Figure 5). Using the VILL antibody, immunoblots of embryos injected with VILL-MO and SC at 6 dpf revealed a single immunoreactive band at 100 kDa (Figure 5H). The abundance of VILL in the VILL-MO-injected group was approximately 3-fold lower than in the SC-injected group (Figure 5H). The apical surfaces of ionocytes were investigated by SEM on the posterior trunks of embryos injected with VILL-MO and SC at 6 dpf (Figure 6A,B). The apical surfaces of embryonic ionocytes were found to exhibit smaller apical and fewer protrusions in the VILL-MO group than the SC group. In addition, there were numerous protrusions in the apical surfaces of pavement cells of SC-injected embryos compared to those of the VILL-MO group (Figure 6C,D).

## Discussion

Lee et al. [10] reported that cytoskeletal filaments were rich in the cell cortex of tilapia ionocyte with microvilli. In the FW medaka, SEM and TEM showed numerous finger-like protrusions extending from the cell membrane in the apical openings of the ionocytes. The finger-like protrusions exhibited parallel bundles of cytoskeletal filaments. The parallel bundles of filaments were originated from the terminal web with web-like cytoskeletal filaments in the cell cortex of the medaka ionocyte. According to the known characteristics of microvilli in the epithelial cells of mammals [14-16], the filament-rich protrusions of medaka ionocytes were microvilli. Among the ionocytes of the brackish medaka acclimated to different salinities, abundant microvilli were found on the apical surfaces of the lower-salinity groups. Microvilli are densely packed on the apical surfaces of the ionocytes responsible for ion absorption in FW fishes [5,8-13]. Similar to the other euryhaline teleosts exposed to hypoosmotic challenges, the gill ionocytes of the brackish medaka enlarged their apical surface area with microvilli when transferred from SW to FW [13]. Therefore, the plasticity in the formation of ionocyte microvilli was hypoosmotic-dependent in the medaka gills.

Like villin 1, the predicted VILL of the medaka also exhibited six gelsolin segments and one headpiece domain. These domains indicated that medaka VILL could bind with G- and F-actin to play the roles of actin-capping, -severing, -nucleating and -bundling [24]. We surveyed databases of the 11 fish species in the Ensembl Genome Browser (Table 1) and compared the fish genomes [36]. According to the phylogenetic tree, VILL of the brackish medaka was more similar to the other VILL orthologs of the teleosts. In addition, the agnathan, an early vertebrate, only exhibited VILL. The phylogenetic tree presented in this study suggests that VILL is an ancestor of vertebrate villin 1.

By RT-PCR analysis of 11 organs, we found that *Odvill* was expressed mainly in the gill, kidney, intestine, ovary, and testis. In humans, *villin 1* is expressed in the intestine, colon, kidney, and testis [37]. Among these tissues, the human ileum exhibited the highest abundance of *villin 1*. Wang et al. [38] reported the expression of the *villin 1* gene in the intestine of the zebrafish. In the brackish medaka *Odvill* would be expected to conduct some critical functions of the enterocytes of the intestine because of lacking *villin 1*. Furthermore, this study is the first to identify the expression of the *Odvill* mRNA in the gill, the respiratory and ionoregulatory organ of the teleost.

Ultrastructural observations revealed that the apical openings of gill ionocytes in the FW-acclimated medaka had microvilli. In different epithelial cells of mammals, the apical surfaces with various types of microvilli are the major locations of villin 1 protein [23,24,39-41]. Villin 1 organizes the actin filaments for the formation of microvilli in the apical region of mammalian enterocytes [25,26]. In the chicken, villin 1 is also expressed in the microvilli of enterocytes [21,42]. On the other hand, villin 1 protein is located in the apical microvilli of enterocytes and taste cells of the amphibian [43,44]. In the brackish medaka, gill ionocytes expressed NKA on the basolateral membrane [13,32-35]. The images of whole-mount double immunofluorescence staining revealed that VILL protein was localized in the apical region of the NKA immunoreactive (NKA-IR) cells in gills of the FW fish but not in the SW group. Additionally, because a monoclonal antibody, T4, has been successfully used to label the apical Na^+^, Cl^-^ cotransporter in gill ionocytes of FW-acclimated fish [33,45], the present study used double immunofluorescence staining with the T4 antibody to illustrate that VILL was localized at the apical region of absorptive ionocytes. Some VILL-immunoreactive (IR) cells, however, exhibited no apical T4-IR signals in the FW medaka. Hiroi et al. [45] reported that type III (apical T4-IR negative) and type II (apical T4-IR positive) ionocytes are involved in the absorption of Na^+^ and Cl^-^, respectively, in gills of tilapia when exposed to hypoosmotic environments. The results revealed that the apical VILL expressed in the absorptive ionocytes of medaka gills. Our TEM observations demonstrated that gill ionocytes exhibited more cytoskeletal filaments in the region of the cell cortex of FW ionocytes. Therefore, we speculated that the VILL protein would accumulate in the cytoskeleton-rich region so that it could participate in the formation of microvilli in the FW ionocytes. Across diverse taxa and broad evolutionary distances, epithelial cells express conserved molecules for the formation of microvilli. The antibody of VILL would be a relevant marker to identify the apical region of the absorptive ionocytes in gills of teleosts.

Based on our observation of superior salinity tolerance of the euryhaline medaka [13,33], the correlation between VILL expression and the formation of microvilli in gill ionocytes was investigated in chronic and acute salinity-exposure experiments. Quantification of branchial *Odvill* mRNA revealed the hypoosmotic-dependent expression in gills of medaka acclimated to SW, 50% SW, or FW. The VILL antibody detected the major and minor immunoreactive bands at 100 kDa and 90 kDa, respectively, in the protein lysates of medaka gills. Immunoblots showing two bands have been reported in frog embryos labeled with an anti-frog villin 1 antibody [43]. Based on the predicted weight of 96–98 kDa from its deduced amino acid sequence, the larger band was most likely intact VILL protein of the brackish medaka. In the mouse kidney, the villin 1 protein (92 kDa) is degraded to fragments with lower molecular weights of 83 kDa and 53 kDa by meprins [46]. We proposed that the minor bands of VILL immunoblots might be degraded forms of medaka VILL in the gill ionocytes with microvilli. Combining the amounts of these two immunoreactive bands, the expression of VILL protein showed a similar pattern to that of *Odvill* mRNA and corresponded to the signals of immunofluorescence staining. The formation of ionocyte microvilli was correlated with the expression levels of VILL protein in gills of the medaka when acclimated to environments with decreasing salinities. On the other hand, time-course experiments indicated the increased gene and protein expression of VILL in gills of the SW medaka after being transferred to FW. In line with the typical model of protein synthesis, the mRNA level of VILL increased more quickly than the protein level after transfer. The highest level of VILL protein was detected in the medaka transferred from SW to FW for 4 days. In fish at the same time-point, microvilli were observed on the apical surface of gill ionocytes [13]. The occurrence of microvilli in ionocytes coincided with increasing levels of VILL protein in gills of the brackish medaka upon hypoosmotic challenge. When villin 1 was overexpressed in the fibroblasts, numerous microvilli were formed on their plasma membrane [27,28]. Therefore, the results of the chronic and acute salinity-exposure experiments in this study confirm that decreasing environmental salinity can induce the expression of VILL at both mRNA and protein levels in gill ionocytes of the euryhaline medaka. Because the constant expression of *villin 1* mRNA is useful to measure the variation in the mammalian enterocyte content of biopsies [47], the elevated expression of *Odvill* mRNA implies that a specific promoter in the upstream region of *Odvill* in the brackish medaka is induced in response to decreasing environmental salinity. The expression of VILL protein was hypoosmotic-dependent and correlated with the formation of microvilli on the apical surfaces of gill ionocytes. To our knowledge, this study is the first to illustrate the expression of fish VILL protein associated with the changes in cellular morphology for ionoregulation in gills.

The antisense morpholino oligonucleotide (MO) is a popular and reliable approach for gene knockdown to demonstrate the *in vivo* functions of target genes using the embryos of the zebrafish and Japanese medaka [48,49]. We developed a microinjection system of MO knockdown in the transparent embryo of the brackish medaka to examine the cellular function of VILL protein in the present study. Ionocytes were found in the epithelia of the yolk sac of the Japanese medaka embryos as early as 2 dpf. At 4 dpf, the ionocytes of the trunk epithelia appeared in the embryos [50]. Similar to the gill ionocytes, the apical openings of the ionocytes remodel the epithelial cell morphology of the embryonic trunk and yolk sac of the tilapia [51], sea bass [52], and zebrafish [53] in the early developmental stages as the ambient ion concentrations change. Recent studies have performed MO knockdown of ion transporters in embryonic ionocytes to elucidate the ionoregulatory functions of proteins in fish gills [53-55]. The duration of MO efficiency in the inhibition of protein translation is 7–10 days in the embryos of the zebrafish and Japanese medaka [48,49]. In the developmental stages of the brackish medaka, numerous apical openings of ionocytes were observed in the trunk of FW-incubated embryos at 6 dpf. The embryonic ionocytes exhibited basolateral and apical patterns of VILL-IR signals (compared to NKA) in the trunks that were different from those in the ionocytes of adult gills, which had only apical signals. The results indicated that the embryonic ionocytes could express VILL for the formation of apical microvilli. According to the results of immunoblotting and immunostaining, the efficiency and specificity of VILL-MO blocked the translation initiation of *Odvill* mRNA and thus reduced the expression of VILL protein in the ionocytes of the microinjected embryos. Differentiation of ionocytes, however, might not have been affected by the VILL-MO treatment in the posterior trunk of the embryos at 6 dpf. On the other hand, SEM observation was used to verify the disrupting effects on the cellular morphologies of the ionocytes after VILL-MO knockdown in the embryos at 6 dpf. The apical surfaces of ionocytes without VILL expression showed smaller openings with fewer protrusions in the morphants compared to the SC-injected group. In addition, circular patterns of VILL signals and numerous apical microridges were found in the pavement cells (PVCs) adjacent to apical openings of the embryonic ionocytes, but these were not found in the PVCs of the gills of adult medaka. The apical surfaces of PVCs lacked microridges in the morphants. In a mammalian intestinal cell line, the suppression of villin 1 impairs the formation of microvilli [29]. Therefore, the results of MO knockdown indicate that the fish VILL protein plays an important role in remodeling cellular morphology, including the formation of microvilli and microridges on the apical surfaces of epithelial cells *in vivo*.

## Conclusions

In the brackish medaka when environmental salinity is decreasing, the hypoosmotic-dependent microvilli are enhanced on the apical surfaces of gill ionocytes. The phylogenetic tree and functional domains suggest that VILL is the homolog of villin 1 in fishes. This study is the first to show that the expression of VILL, an actin-regulatory protein, is associated with the formation of microvilli in the absorptive ionocytes of a euryhaline fish. Loss-of-function experiments show that the distribution of VILL may represent the molecular link between the cytoskeleton organization and cellular morphology. Consequently, the availability of VILL protein will be helpful in improving our understanding of the molecular evolution of microvilli in relation to the functional morphologies of the absorptive ionocytes during hypoosmotic adaptation in aquatic vertebrates.

## Methods

### Experimental animals and environments

The brackish medaka (*Oryzias dancena*; 2.5 ± 0.3 cm) was reared in 50% SW. The water was continuously circulated through fabric-floss filters and partially refreshed every two weeks and temperature was held at 28 ± 1°C. Fish received a constant photoperiod cycle of 14:10 hrs light: dark cycle and were fed a daily diet of commercial pellets. The facilities and protocols for experimental animals were approved by the Institutional Animal Care and Use Committee of the National Chung Hsing University (IACUC approval no. 96–48 to THL). Following the experiment, fish were not fed and were anaesthetized with MS-222 (100–200 mg/l) before sampling. For chronic (long-term) experiments, the fish were acclimated to FW, 50% SW (15%), or SW (35%) for at least one month. On the other hand, SW-acclimated medaka was transferred directly to FW for acute (short-term) experiments. The FW-exposed groups were sampled at 1/2, 1, 2, 4, or 7 days.

### Electron microscopic observations

The gills or embryos were fixed at 4°C in a fixative consisting of 5% (v/v) glutaraldehyde and 4% (w/v) paraformaldehyde in 0.1 M phosphate buffer (PB, pH 7.2) for 12 hrs. Fixed samples were post-fixed with 1% (w/v) osmium tetroxide in 0.1 M PB for 1.5 hrs. After post-fixation, the samples were rinsed in PB and dehydrated in ascending concentrations of ethanol. For observation in the scanning electron microscope (SEM, JSM-6700 F, JEOL, Tokyo, Japan), samples were prepared by our previous method [13]. For observation in the transmission electron microscope (JEM-1400, JEOL), the dehydrated gills were transferred to propylene oxide and embedded in Spurr’s resin. Ultrathin sections (75 nm) of gill filaments were cut with MTX Ultramicrotome (RMC, Tucson, AZ, USA and mounted on grids. The sections were stained with uranyl acetate and lead citrate before observation.

### Double immunofluorescence staining

Gills of medaka were fixed in the neutral formalin (pH 7.2) in 4°C for 3 hrs. After washing in phosphate buffer saline (PBS), the samples of whole-mount immunofluorescence staining were permeated with methanol for 30 min at -20°C. The samples were then stored in the methanol at -20°C before performing the following experiments. Whole-mount samples were rehydrated by rinsing with PBS three times. Subsequently, the samples were incubated in 5% bovine serum albumin (Sigma, St. Louis, MO, USA) at room temperature for 30 min. Samples were then incubated with the rabbit polyclonal VILL antibody (1: 5000 dilution) at room temperature for 2 hrs. Samples were then washed with PBS, incubated with the secondary antibody (Dylight-488 goat anti-rabbit IgG) at room temperature for 1 hr and washed again with PBS. After the first staining, samples were incubated with the mouse monoclonal antibody (α5, 1:200 or T4, 1:100) for 2 hrs at room temperature. Samples were then washed and incubated with the secondary antibody (Dylight-549 goat anti-mouse IgG) at room temperature for 1 hr followed by several PBS washes. Samples were then mounted in 50% glycerol with PBS and examined using a fluorescent microscope (BX50, Olympus, Tokyo, Japan) with the Olympus DP72 CCD camera or a laser scanning confocal microscope (FV1000, Olympus).

### Gene identification

The template of cDNA for gene cloning and SMART RACE cDNA amplification kit (Clontech, Palo Alto, CA, USA) were described in our previous study [33]. Amino acid sequences for villin and VILL from different organisms (Table 1) were aligned by CLUSTALW and a phylogenetic tree was constructed using MEGA 4.1. For tissue distribution of gene expression, PCR amplification (28 cycles) was done by the Hot start EX-Taq polymerase (Takara, Shiga, Japan). β-actin was used as an internal control for all tissues. The sequences of the primers used in the gene identification were listed in the Table 2.

**Table 2:** **Primer sequences used for cDNA cloning, RACE, RT-PCR (for tissue distribution) and quantitative real-time PCR of the *Odvill *** **gene of the brackish medaka**

**Primer name**	**Primer sequence (5′ to 3′)**
*Odvill*-F	CCTGCAGATATGGACCATCAA
*Odvill*-R	CAAGTCATTCTGACGCCACTT
*Odvill*-RACE-F	ACACGCCAACAAGTCTGAAACAACAG
*Odvill*-RACE-R	GGACGTTGTAGGAGTTTGTGTCAACG
*Odvill*-RT-F	CAGCTTCAACAACGGAGACA
*Odvill*-RT-R	ACCGTCACATCTCCAGAAGC
*Odvill*-QPCR-F	GTTCAAGAAGTGGCCACACA
*Odvill*-QPCR-R	TTGCTCTGTTCATGGCTTCTT
β-actin-F	CTGGACTTCGAGCAGGAGAT
β-actin-R	AGGAAGGAAGGCTGGAAGAG

### Real-time PCR

The *Odvill* mRNA of gills was detected by the SYBR Green PCR Master Mix (Bio-Rad Laboratories, Hercules, CA, USA) and quantified with the Mini Opticon real-time PCR system (Bio-Rad). The *Odvill* mRNA values were normalized using the expression of the β-actin mRNA from the same DNA samples. One identical cDNA sample from the 50% SW-acclimated fish was used as the internal control among different groups. For each unknown sample, the comparative Ct method with the formula 2^-[(Ct_*Odvill*,n_-Ct_β-actin,n_)-(Ct_*Odvill*,c_-Ct_β-actin,c_)] was used to obtain the corresponding *Odvill* and β-actin values, where Ct corresponded to the threshold cycle number.

### Protein extraction and western immunodetection

According to the method of Kang et al. [13], homogenates of medaka gills or embryos were prepared. Aliquots containing 20 μg of homogenates were heated at 65°C for 25 min and fractionated by electrophoresis on SDS-containing 7.5% polyacrylamide gels. Transferred PVDF blots were incubated with VILL polyclonal antibody (1:200,000 dilution) or β-actin monoclonal antibody (1:10,000 dilution; 8226, Abcam, Cambridge, UK) for 2 hrs, followed by a 1-hr reaction with horseradish peroxidase-conjugated goat anti-mouse or anti-rabbit IgG (Pierce, Rockford, IL, USA). Blots were developed using the SuperSignal West Pico Detection Kit (#34082, Pierce) and analyzed with Image Lab software version 3.0 (Bio-Rad) after densitometric scanning of membranes using a ChemiDoc XRS+ (Bio-Rad). Results were converted to numerical values to compare relative intensities of immunoreactive bands.

### Microinjection of morpholino oligonucleotides

Morpholino oligonucleotides (MOs) of antisense *Odvill* were designed and manufactured by Gene Tools (Philomath, OR, USA). The VILL-MO sequence was 5′-TGTTCGGAAAGTCATCCTTCATCAT-3′. This morpholino overlapped the start codon (underlined) and acted as a translational blocker. Gene Tools’ standard control (SC) morpholino (5′-CCTCTTACCTCAGTTACAATTTATA-3′) was used as a morpholino injection control. Morpholinos were dissolved to 0.5 mM injection stocks in RNase-free water. The microinjection system consisted of an Olympus SZ60 stereomicroscope, an air-pressure-driven microinjector (IM-300; Narishige, Tokyo, Japan), and a three-axis micromanipulator (M-152, Narishige). The fertilized eggs of FW-acclimated medaka at the one- or two-cell stage were collected for operation with the microinjection system in a protocol modified from Kinoshita et al. [49]. The injection volumes of VILL-MO and SC ranged from 2 to 5 nl. Injection pressure was varied to compensate for variable needle opening size to ensure an appropriate injection volume. The injected eggs were incubated in FW at 28±1°C. The morphologies of developing embryos were investigated and recorded for 6 days after fertilization (dpf). The 6 dpf embryos were collected in a microcentrifuge and stored in -80°C for Western blotting. For immunofluorescence staining and SEM, the embryos were fixed after performing the dechorionation procedure.

### Bioinformatics and statistical analysis

The open reading frame of *Odvill* was predicted with the ORF Finder (http://www.ncbi.nlm.nih.gov/gorf/gorf.html). Six gelosolin and one head piece domains of the medaka VILL were analyzed in the website of Conserved Domains and Protein Classification (http://www.ncbi.nlm.nih.gov/Structure/cdd/cdd.shtml). Values were expressed as the means ± S.E.M. (the standard error of the mean). Results were compared using Student’s t-test or one-way ANOVA followed by Tukey’s pairwise method. The significance level was set as p < 0.05.

## Abbreviations

dpf: Day post-fertilization; FW: Fresh water; HE: Hematoxylin-eosin; MO: Morpholino oligonucleotide; NKA: Na^+^, K^+^-ATPase; NKA-IR cells: NKA-immunoreactive cells; SC: Standard control; SEM: Scanning electron microscopy; SW: Seawater; TEM: Transmission electron microscopy; VILL: Villin 1-like protein.

## Competing interests

The authors have no commercial, proprietary or financial interest in any companies or products described in this article.

## Authors’ contributions

CKK and THL conceived the study. CKK and THL discussed and designed the experiments. CKK performed the experiments and wrote the manuscript. Both authors read and approved the final manuscript.

## Supplementary Material

Additional file 1 Additional figures supporting the data analysis. Click here for file

## References

[B1] Marshall WS Na^+^, Cl^-^, Ca^2+^ and Zn^2+^ transport by fish gills: retrospective review and prospective synthesis J Exp Zool 2002 293 264 283 10.1002/jez.10127 12115901

[B2] Hirose S Kaneko T Naito N Takei Y Molecular biology of major components of chloride cells Comp Biochem Physiol B 2003 136 593 620 10.1016/S1096-4959(03)00287-2 14662288

[B3] Evans DH Piermarini PM Choe KP The multifunctional fish gill: Dominant site of gas exchange, osmoregulation, acid–base regulation, and excretion of nitrogenous waste Physiol Rev 2005 85 97 177 10.1152/physrev.00050.2003 15618479

[B4] Hwang PP Lee TH New insights into fish ion regulation and mitochondrion-rich cells Comp Biochem Physiol A 2007 148 479 497 10.1016/j.cbpa.2007.06.416 17689996

[B5] Kaneko T Watanabe S Lee KM Functional morphology of mitochondrion-rich cells in euryhaline and stenohaline teleosts Aqua BioSci Monogr 2008 1 1 62

[B6] Wilson JM Farrell AP Enciclopedia of fish physiology: from genome to environment 2011 Waltham: Academic Press 1381 1388

[B7] Hwang PP Lee TH Lin LY Ion regulation in fish gills: recent progress in the cellular and molecular mechanisms Am J Physiol 2011 301 R28 R47 10.1152/ajpregu.00047.2011 21451143

[B8] Edwards SL Marshall WS McCormick SD, Farrell AP, Brauner CJ, New Y, McCormick SD, Farrell AP, Brauner CJ Fish Physiology: Euryhaline Fishes 2013 Waltham: Academic Press 21 33

[B9] Hossler FE Musil G Karnaky KJ Epstein FH Surface ultrastructure of the gill arch of the killifish, *Fundulus heteroclitus*, from seawater and fresh water, with special reference to the morphology of apical crypts of chloride cells J Morphol 1985 185 377 386 10.1002/jmor.1051850309 4057266

[B10] Lee TH Hwang PP Lin HC Huang FL Mitochondria-rich cells in the branchial epithelium of the teleost, *Oreochromis mossambicus*, acclimated to various hypotonic environments Fish Physiol Biochem 1996 15 513 523 10.1007/BF01874924 24194359

[B11] Perry SF The chloride cell: structure and function in the gills of freshwater fishes Annu Rev Physiol 1997 59 325 347 10.1146/annurev.physiol.59.1.325 9074767

[B12] Katoh F Kaneko T Short-term transformation and long-term replacement of branchial chloride cells in killifish transferred from seawater to freshwater, revealed by morphofunctional observations and a newly established ‘time-differential double fluorescent staining technique J Exp Biol 2003 206 4113 4123 10.1242/jeb.00659 14555751

[B13] Kang CK Yang WK Lin ST Liu CC Lin HM Chen HH Cheng CW Lee TH Hwang PP The acute and regulatory phases of time-course changes in gill mitochondrion-rich cells of seawater-acclimated medaka (*Oryzias dancena*) when exposed to hypoosmotic environments Comp Biochem Physiol A 2013 164 181 191 10.1016/j.cbpa.2012.08.010 22960413

[B14] Bray D Heath J Moss D The membrane-associated “cortex” of animal cells: its structure and mechanical properties J Cell Sci Suppl 1986 20 71 88 3528202 10.1242/jcs.1986.supplement_4.5

[B15] Alberts B Johnson A Lewis J Raff M Roberts K Walter P Molecular biology of the cell The cytoskeleton 2002 New York: Garland Science 907 982

[B16] Revenu C Athman R Robine S Louvard D The co-workers of actin filaments: from cell structures to signals Nat Rev Mol Cell Biol 2004 5 635 646 10.1038/nrm1437 15366707

[B17] Maina JN A study of the morphology of tile gills of an extreme alkalinity and hyperosmotic adapted teleost *Oreochromis alcalicus grahami* (Boulenger) with particular emphasis on the ultrastructure of the chloride cells and their modifications with water dilution Anat Embryol 1990 181 83 98 2305972 10.1007/BF00189731

[B18] Cioni C De Merich D Cataldi E Sataudella S Fine structure of chloride cells in freshwater-and seawater-adapted *Oreochromis niloticus* (Linnaeus) and *Oreochromis mossambicus* (Peters) J Fish Biol 1991 39 197 209 10.1111/j.1095-8649.1991.tb04356.x

[B19] Pisam M Lemoal C Auperm B Prunet P Rambourg A Apical structures of “mitochondria-rich” α and β cells in euryhaline fish gill: their behaviour in various living conditions Anat Rec 1995 241 13 24 10.1002/ar.1092410104 7879919

[B20] Wong CK Chan DK Isolation of viable cell types from the gill epithelium of Japanese eel *Anguilla japonica* Am J Physiol 1999 276 R363 R372 9950913 10.1152/ajpregu.1999.276.2.R363

[B21] Bretscher A Weber K Villin: the major microfilament‒associated protein of the intestinal microvillus Proc Natl Acad Sci U S A 1979 76 2321 2325 10.1073/pnas.76.5.2321 287075 PMC383592

[B22] Friederich E Vancompernolle K Louvard D Vandekerckhove J Villin function in the organization of the actin cytoskeleton. Correlation of *in vivo* effects to its biochemical activities *in vitro* J Biol Chem 1999 274 26751 26760 10.1074/jbc.274.38.26751 10480879

[B23] Khurana S Structure and function of villin Adv Mol Cell Biol 2006 37 89 117

[B24] Khurana S George SP Regulation of cell structure and function by actin-binding proteins: Villin’s perspective FEBS Lett 2008 582 2128 2139 10.1016/j.febslet.2008.02.040 18307996 PMC2680319

[B25] Hampton CM Liu J Taylor DW DeRosier DJ Taylor KA The 3D structure of villin as an unusual F-actin crosslinker Structure 2008 16 1882 1891 10.1016/j.str.2008.09.015 19081064 PMC2782859

[B26] Brown JW McKnight CJ Molecular model of the microvillar cytoskeleton and organization of the brush border PLoS One 2010 5 e9406 10.1371/journal.pone.0009406 20195380 PMC2827561

[B27] Friederich E Pringault E Arpin M Louvard D From the structure to the function of villin, an actin-binding protein of the brush border Bioessays 1990 12 403 408 10.1002/bies.950120902 2256904

[B28] Friederich E Huet C Arpin M Louvard D Villin induces microvilli growth and actin redistribution in transfected fibroblasts Cell 1989 59 461 475 10.1016/0092-8674(89)90030-5 2680107

[B29] de Beauregard MA C Pringault E Robine S Louvard D Suppression of villin expression by antisense RNA impairs brush border assembly in polarized epithelial intestinal cells EMBO J 1995 14 409 421 7859732 10.1002/j.1460-2075.1995.tb07017.x PMC398099

[B30] Inoue K Takei Y Diverse adaptability in *Oryzias* species to high environmental salinity Zool Sci 2002 19 727 734 10.2108/zsj.19.727 12149572

[B31] Inoue K Takei Y Asian medaka fishes offer new models for studying mechanisms of seawater adaptation Comp Biochem Physiol B 2003 136 635 645 10.1016/S1096-4959(03)00204-5 14662290

[B32] Kang CK Tsai SC Lee TH Hwang PP Differential expression of branchial Na^+^/K^+^-ATPase of two medaka species, *Oryzias latipes* and *Oryzias dancena*, with different salinitytolerances acclimated to fresh water, brackish water and seawater Comp Biochem Physiol A 2008 151 566 575 10.1016/j.cbpa.2008.07.020 18692588

[B33] Kang CK Tsai HJ Liu CC Lee TH Hwang PP Salinity-dependent expression of a Na^+^, K^+^, 2Cl^-^ cotransporter in gills of the brackish medaka *Oryzias dancena*: a molecular correlate for hyposmoregulatory endurance Comp Biochem Physiol A 2010 157 7 18 10.1016/j.cbpa.2010.05.013 20576485

[B34] Kang CK Tsai SC Lin ST Lee TH Hwang PP Cystic fibrosis transmembrane conductance regulator (CFTR): an apical marker protein of ionocytes for identifying hypoosmoregulation in gills of the euryhaline medaka, *Oryzias dancena* Zool Stud 2012 51 1270 1281

[B35] Yang WK Kang CK Chang CH Hsu AD Lee TH Hwang PP Expression profiles of branchial FXYD proteins in the brackish medaka *Oryzias dancena*: a potential saltwater fish model for studies of osmoregulation PLoS One 2013 8 e55470 10.1371/journal.pone.0055470 23383199 PMC3561181

[B36] Venkatesh B Evolution and diversity of fish genomes Curr Opin Genet Dev 2003 13 588 592 10.1016/j.gde.2003.09.001 14638319

[B37] Zhu LJ Altmann SW mRNA and 18S-RNA coapplication-reverse transcription for quantitative gene expression analysis Anal Biochem 2005 345 102 109 10.1016/j.ab.2005.07.028 16139233

[B38] Wang Z Du J Lam S Mathavan S Matsudaira P Gong Z Morphological and molecular evidence for functional organization along the rostrocaudal axis of the adult zebrafish intestine BMC Genomics 2010 11 392 10.1186/1471-2164-11-392 20565988 PMC2996925

[B39] Bretscher A Osborn M Wehland J Weber K Villin associates with specific microfilamentous structures as seen by immunofluorescence microscopy on tissue sections and cells microinjected with villin Exp Cell Res 1981 135 213 219 10.1016/0014-4827(81)90313-X 7026267

[B40] Robine S Huet C Moll R Sahuquillo-Merino C Coudrier E Zweibaum A Louvard D Can villin be used to identify malignant and undifferentiated normal digestive epithelial cells? Proc Natl Acad Sci U S A 1985 82 8488 8492 10.1073/pnas.82.24.8488 3909146 PMC390941

[B41] Hofer D Drenckhahn D Identification of brush cells in the alimentary and respiratory system by antibodies to villin and fimbrin Histochemistry 1992 98 237 242 10.1007/BF00271037 1459863

[B42] Heintzelman MB Mooseker MS Assembly of the brush border cytoskeleton: changes in the distribution of microvillar core proteins during enterocyte differentiation in adult chicken intestine Cell Motil Cytoskeleton 1990 15 12 22 10.1002/cm.970150104 2403846

[B43] Heusser S Colin S Figiel A Huet C Keller JM Pornet P Robine S Vandamme J Vandekerckhove J Dauca M Amphibian intestinal villin: isolation and expression during embryonic and larval development J Cell Sci 1992 103 699 708 1478966 10.1242/jcs.103.3.699

[B44] Yoshie S Kumakura M Toyoshima K Villin is a possible marker of receptor cells in frog taste organs Histochem Cell Biol 2003 119 447 450 10.1007/s00418-003-0533-4 12768287

[B45] Hiroi J Yasumasu S McCormick SD Hwang PP Kaneko T Evidence for an apical Na^+^ Cl^-^ cotransporter involved in ion uptake in a teleost fish J Exp Biol 2008 211 2584 2599 10.1242/jeb.018663 18689412

[B46] Ongeri EM Anyanwu O Reeves WB Bond JS Villin and actin in the mouse kidney brush-border membrane bind to and are degraded by meprins, an interaction that contributes to injury in ischemia-reperfusion Am J Physiol 2011 301 F871 F882 10.1152/ajprenal.00703.2010 PMC3191804 21795642

[B47] Lown KS Mayo RR Leichtman AB Hsiao HL Turgeon DK Schmiedlin-Ren P Brown MB Guo W Rossi SJ Benet LZ Watkins PB Role of intestinal P-glycoprotein (*mdr1*) in interpatient variation in the oral bioavailability of cyclosporine Clin Pharmacol Ther 1997 62 248 260 10.1016/S0009-9236(97)90027-8 9333100

[B48] Nasevicius A Ekker SC Effective targeted gene ‘knockdown’ in zebrafish Nat Genet 2000 26 216 220 10.1038/79951 11017081

[B49] Kinoshita M Murata K Naruse K Tanaka M Medaka: Biology, Management, and Experimental Protocols 2009 Iowa: Wiley-Blackwell, Ames 292 293

[B50] Thermes V Lin CC Hwang PP Expression of *Ol-foxi3* and Na^+^/K^+^-ATPase in ionocytes during the development of euryhaline medaka (*Oryzias latipes*) embryos Gene Expr Patterns 2010 10 185 192 10.1016/j.gep.2010.04.001 20388555

[B51] Hiroi J Kaneko T Tanaka M In vivo sequential changes in chloride cell morphology in the yolk-sac membrane of Mozambique tilapia (*Oreochromis mossambicus*) embryos and larvae during seawater adaptation J Exp Biol 1999 202 3485 3495 10574727 10.1242/jeb.202.24.3485

[B52] Varsamos S Diaz JP Charmantier G Blasco C Connes R Flik G Location and morphology of chloride cells during the postembryonic development of the European sea bass, *Dicentrarchus labrax* Anat Embryol 2002 205 203 213 10.1007/s00429-002-0231-3 12107490

[B53] Horng JL Lin LY Huang CJ Katoh F Kaneko T Hwang PP Knockdown of V-ATPase subunit A (atp6v1a) impairs acid secretion and ion balance in zebrafish (*Danio rerio*) Am J Physiol 2007 292 R2068 R2076 10.1152/ajpregu.00578.2006 17272665

[B54] Shih TH Horng JL Hwang PP Lin LY Ammonia excretion by the skin of zebrafish (*Denio rerio*) larvae Am J Physiol 2008 295 C1625 C1632 10.1152/ajpcell.00255.2008 18815227

[B55] Wang YF Tseng YC Yan JJ Hiroi J Hwang PP Role of SLC12A10.2, a Na-Cl cotransporter-like protein, in a Cl uptake mechanism in zebrafish (*Danio rerio*) Am J Physiol 2009 296 R1650 R1660 10.1152/ajpregu.00119.2009 19279294

