# Supplementary material for: Medaka villin 1-like protein (VILL) is associated with the formation of microvilli induced by decreasing salinities in the absorptive ionocytes
Source: Front Zool. 2014 Jan 13;11:2. doi: 10.1186/1742-9994-11-2 (PMC3896669; doi:10.1186/1742-9994-11-2)
Supplement: Additional file 1 — Additional figures supporting the data analysis. [file 1742-9994-11-2-S1.doc]

**
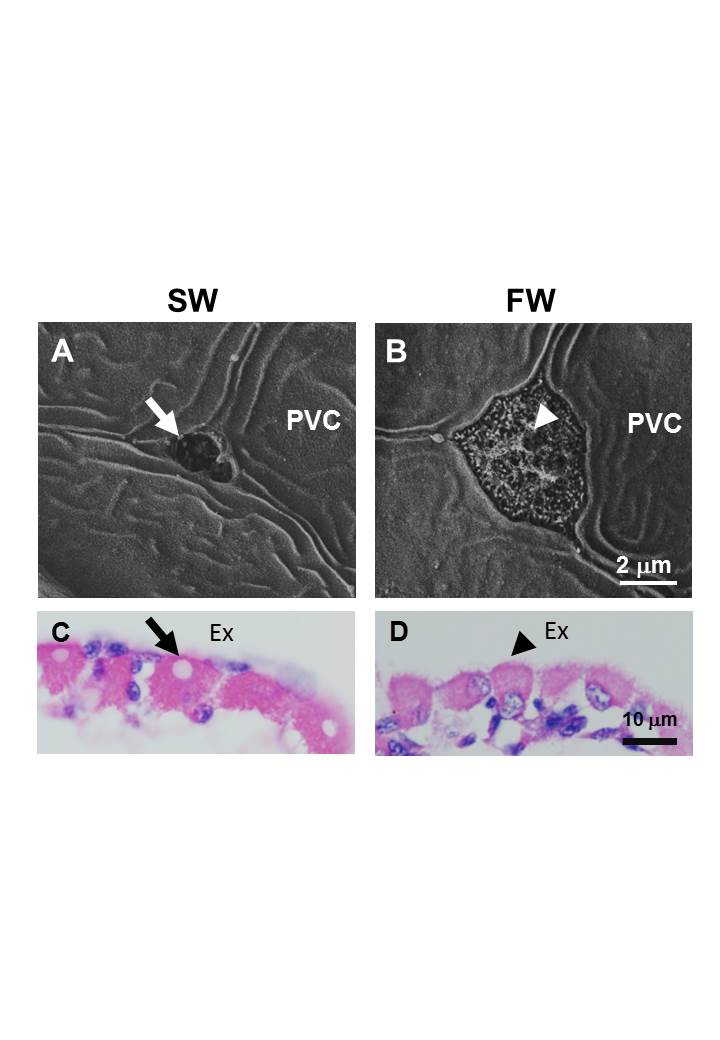
**

**Figure S1** Micrographs of SEM (**A and B**) and hematoxylin/eosin staining of paraffin sections (**C and D**) revealed that the apical surface morphology of SW-type ionocytes exhibited deeply invaginated surfaces with orifices (arrow, **A and C**), while the apical surfaces of FW-type ionocytes had dense microvilli (arrowhead, **B and D**). The ionocytes were eosinophilic cells. Ex, external environment; PVC, pavement cells.

**
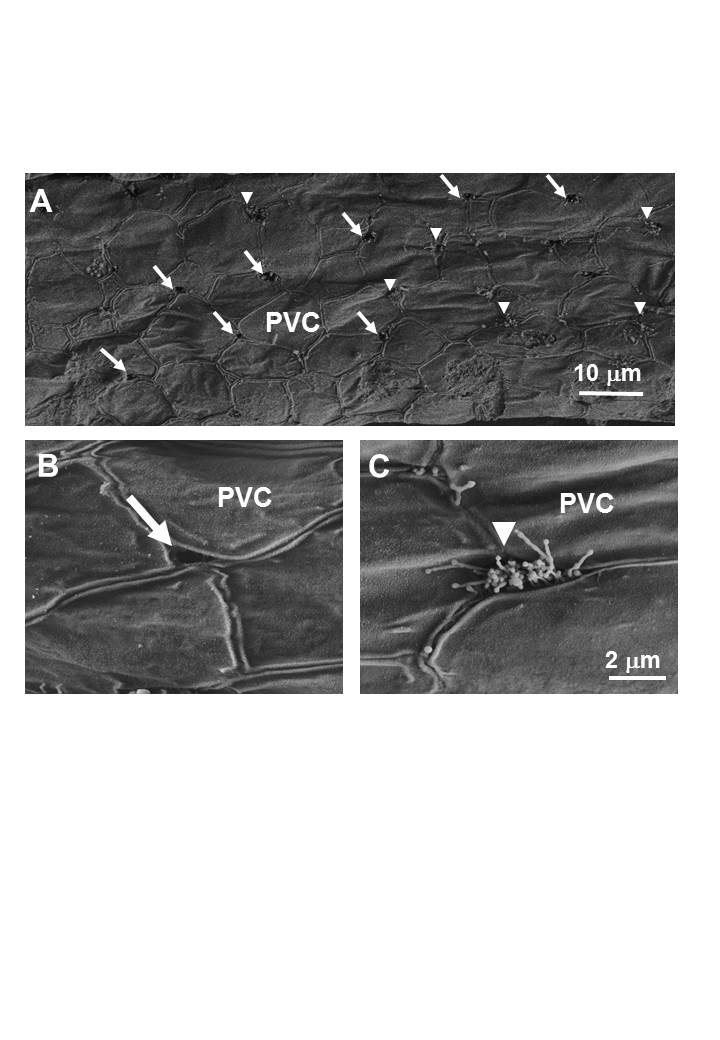
**

**Figure S2** Two types of apical openings of ionocytes were observed in gills of the 50% SW-acclimated brackish medaka (arrows and arrowheads; **A**, **B**, and **C**). The apical openings of 50%-SW-type II (arrowheads, **A** and **C**) ionocytes showed more significant microvilli than 50%-SW-type I (arrows, **A** and **B**). PVC, pavement cells.

**
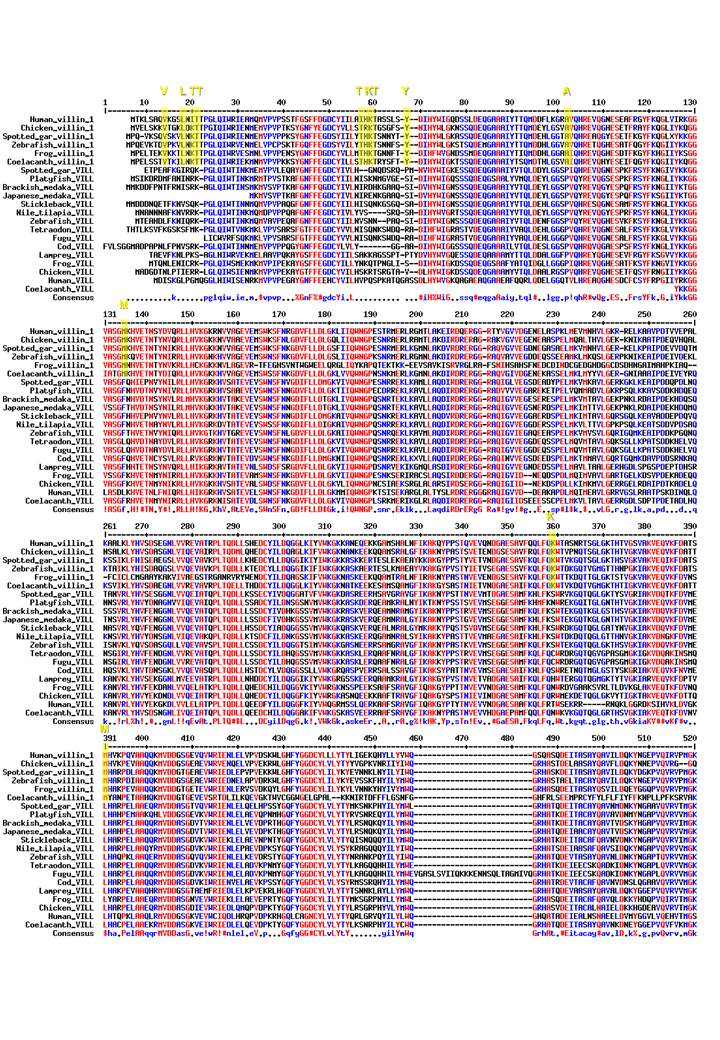
**

**
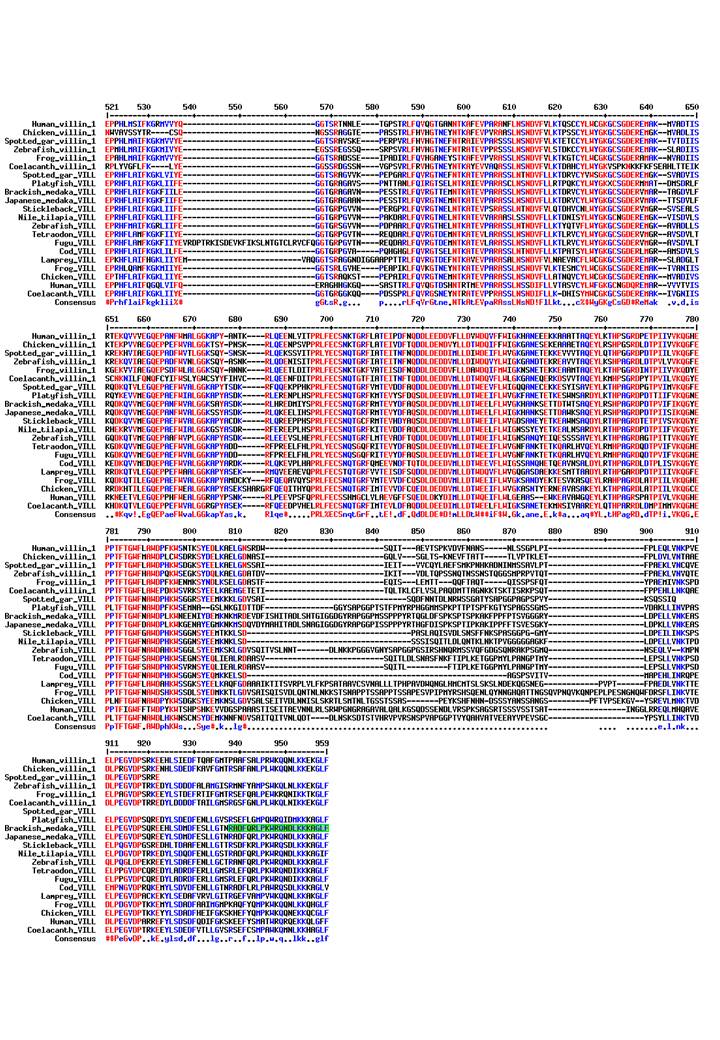
**

**Figure S3** The VILL amino acid sequence of the brackish medaka compared to the villin 1 and VILL sequences of 11 species of fishes. The frog (villin 1: ENSXETP0000003273; VILL: ENSXETP00000056634), chicken (villin 1: ENSGALP00000018624; VILL: ENSGALP00000009244), and human sequences (villin 1: ENSP00000248444; VILL: ENSP00000283713) were introduced to optimize the multiple alignment (http://multalin.toulouse.inra.fr/multalin/multalin.html). All amino acids of villin 1 and VILL that were identical and similar to those of the brackish medaka were shown in red and blue letters, respectively. To distinguish the characteristics between the sequences of villin 1 and VILL, the specific amino sequences of villin 1 were indicated by the yellow words and boxes in the alignment graph. The antigen sequence (ARG-842 to PHE-863) of anti-VILL antibody was marked by the green box.


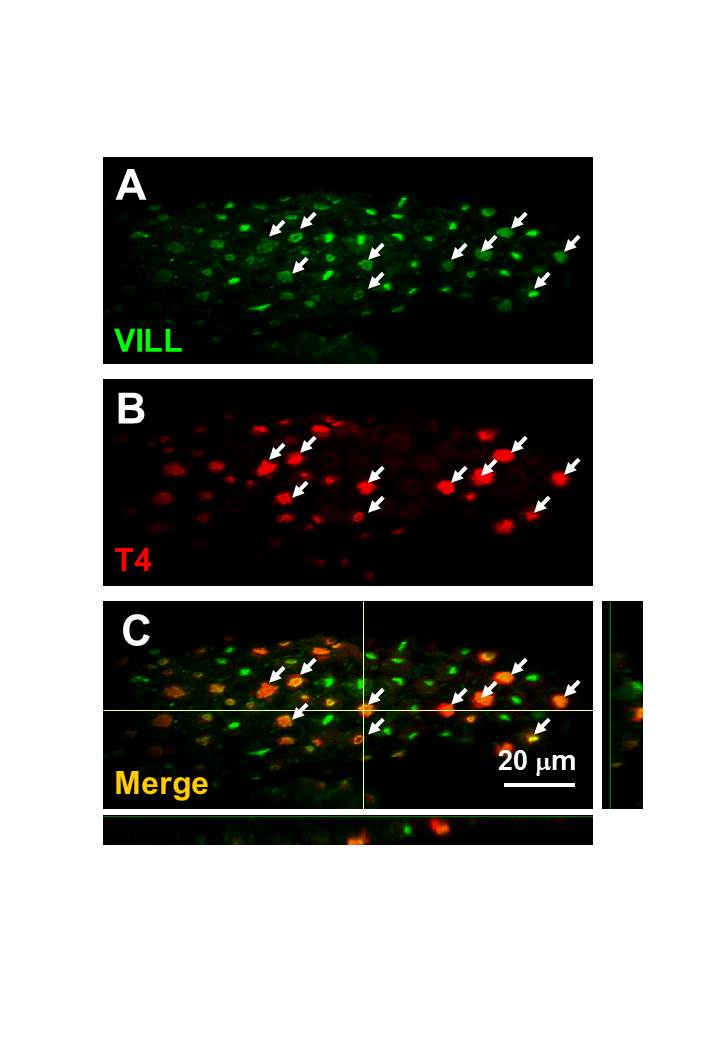


**Figure S4** Confocal micrographs of whole-mount double immunofluorescence staining with the VILL antibody (green; **A**) and the monoclonal T4 antibody (anti-human Na^+^, K^+^, 2Cl^-^ cotransporter; red; **B**) on the afferent side of the gill filament of FW-acclimated brackish medaka. VILL were localized to the apical regions of the FW-type ionocytes in gills of the brackish medaka. In the merged 3D image (**C**), some VILL signals (arrows) were colocalized with apical signals of the T4 antibody. However, some ionocytes with apical VILL protein (green) did not exhibit immunoreactive signals of the T4 antibody.

**
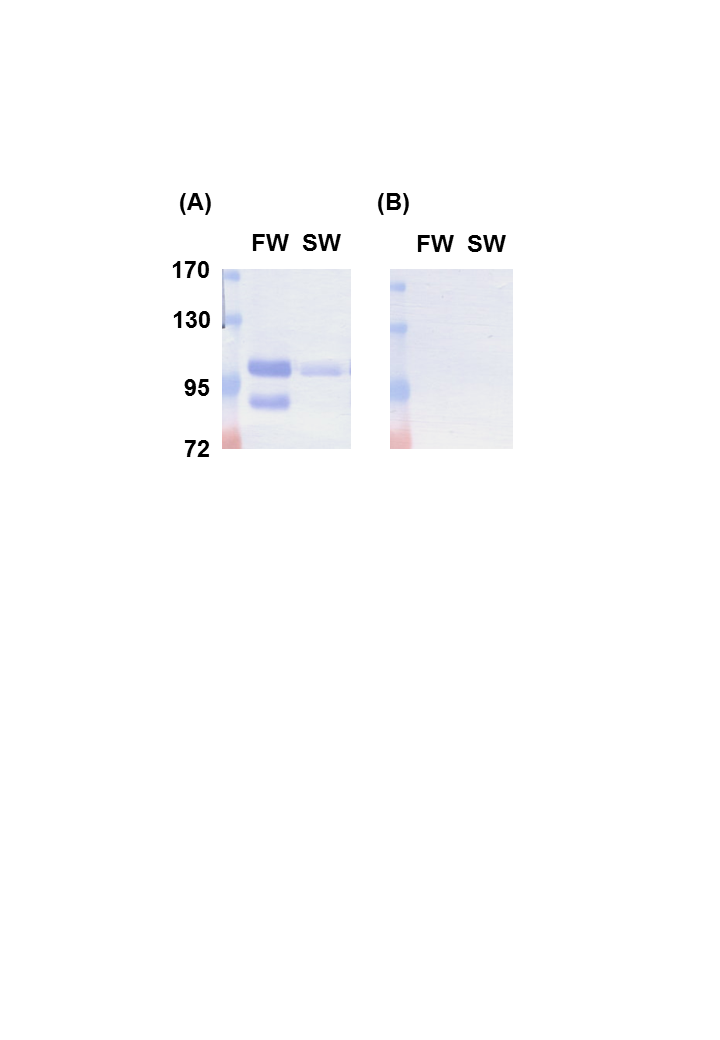
**

**Figure S5** Representative immunoblot and negative-control immunoblot of brackish medaka gills probed with (**A**) a polyclonal antibody against VILL of the brackish medaka and (**B**) the pre-immune serum of rabbit, respectively. The immunoblot of medaka gills probed with the polyclonal antibody revealed two immunoreactive bands, one at 100 kDa in both FW and SW groups and the other band at 90 kDa in the FW group.


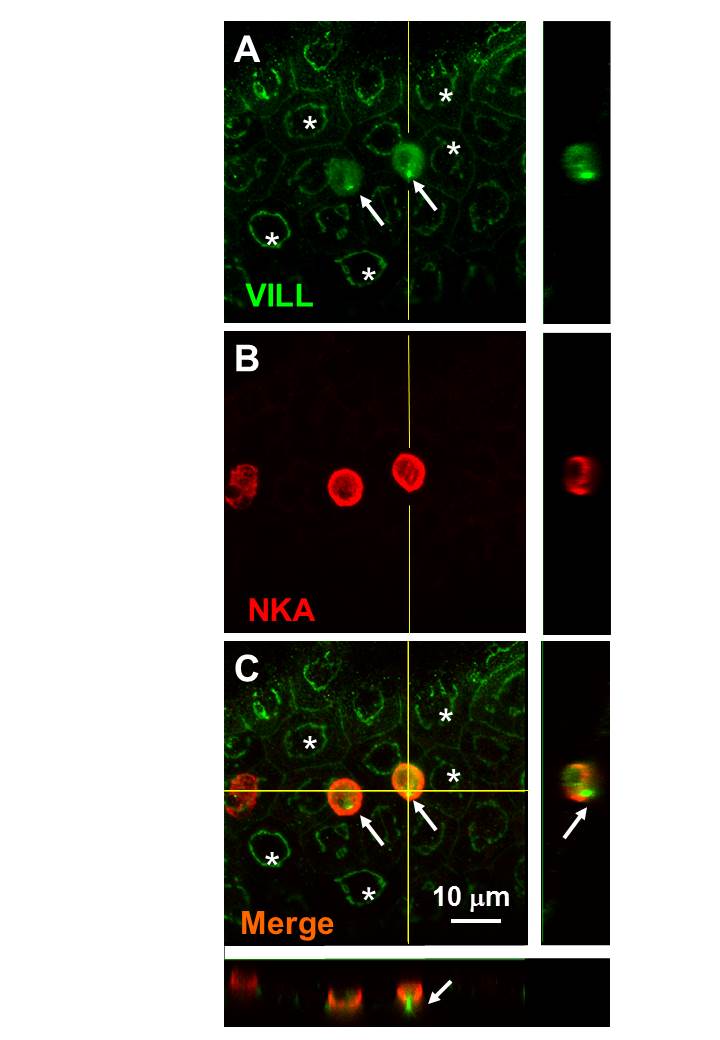


**Figure S6** Confocal micrographs of whole-mount double immunofluorescence staining with anti- VILL (green; **A**) and anti-NKA antibodies (red; **B**) in the posterior trunk of embryos incubated in FW for 6 days after fertilization. The merged images revealed that some NKA-immunoreactive (IR) cells were positive for VILL. The merged 3D images in the X-Z and Y-Z planes (**C**) revealed that the VILL signals were localized to apical regions (arrows) and colocalized with NKA at the basolateral regions of NKA-IR cells. In addition, the circular patterns of VILL signals were also found in the pavement cells (asterisks).
